# Preparation and Characterization of a Multicomponent *Arthrospira platensis* Biomass Hydrolysate with Superior Anti-Hypertensive, Anti-Hyperlipidemic and Antioxidant Activities via Selective Proteolysis

**DOI:** 10.3390/md21040255

**Published:** 2023-04-20

**Authors:** Cristina Otero, Carlos M. Verdasco-Martín

**Affiliations:** Department of Biocatalysis, Institute of Catalysis and Petroleochemistry, Consejo Superior de Investigaciones Científicas-CSIC, 28049 Madrid, Spain

**Keywords:** *Spirulina*, protein extract, functional food, food supplement, digested peptides, anti-hypertensive peptides, essential amino acids, antioxidants, hypocholesterolemic, *Arthrospira*

## Abstract

*Arthrospira platensis* biomass is a sustainable source of bioactive products for the food, cosmetic, and medicine industries. As well as primary metabolites, different secondary metabolites can be obtained via distinct enzymatic degradation of biomass. In this work, different hydrophilic extracts were obtained after treating the biomass with: (i) a serine *endo*-peptidase (Alcalase^®^), (ii) a mixture of amino-, dipeptidyl-, and *endo*-peptidases (Flavourzyme^®^), (iii) a mixture of *endo*-1,3(4)-β-glucanase and an *endo*-1,4-xylanase, and β-glucanase (Ultraflo^®^), and (iv) an *exo*-1,3-glucanase (Vinoflow^®^) (all the enzymes from Novozymes A/S (bagsvaerd, Denmark)); with subsequent extraction of the biocomponents with an isopropanol/hexane mixture. The composition of each aqueous phase extract (in terms of amino acids, peptides, oligo-elements, carbohydrates, and phenols) and their in vitro functional properties were compared. The conditions described in this work using the enzyme Alcalase^®^ permits the extraction of eight distinctive peptides. This extract is 7.3 times more anti-hypertensive, 106 times more anti-hypertriglyceridemic, 26 times more hypocholesterolemic, has 4.4 times more antioxidant activities, and has 2.3 times more phenols, than the extract obtained without any prior enzyme biomass digestion. Alcalase^®^ extract is an advantageous product with potential application in functional food, pharmaceutics, and cosmetics.

## 1. Introduction

Use of microalgae and *Arthrospira* biomass (*Spirulina*) as ingredients in both animal and human foodstuffs has been unceasingly increasing for thousands of years [1]. Moreover, functional products and metabolites are obtained from freshwater and marine *Arthrospira* cyanobacteria for the food, cosmetic, pharmaceutical, and biotechnological industries in developed countries. A wide range of metabolites can be directly extracted from the biomass of these microorganisms, including non-ribosomal and ribosomal peptides, polyketides, terpenes, essential oils, vitamins, antioxidants, oligoelements, etc. [2].

For centuries, freshwater and marine *Arthrospira* has been eaten by humans and nowadays is used as a food additive. It has great potential as a resource of bioproduct for cosmetics and pharmaceuticals. As a protein source, it has similar or superior quality to other vegetable proteins [3], where the nutritional quality of proteins is determined by their contents, proportions, and the bio-availabilities of their amino acids [4]. It provides high amounts of proteins (around 60% of dry biomass, although if it is cultivated under stress conditions, it might produce up to 77% [5]). *Arthrospira platensis* has all the essential amino acids, which are highly used in the livestock industry [6]. *Arthrospira platensis* contains lipids with high polyunsaturated fatty acid content, such as omega-3. It has carbohydrates in a smaller proportion than proteins [7]. Additionally, *Arthrospira platensis* has other interesting biocomponents, such as minerals, antioxidants, vitamins (vitamin B in great proportion), sterols, pigments such as phycocyanin and chlorophyll, etc. *Arthrospira platensis* biomass is classified as a Class A ingredient for its use as dietetic complements by the European Union and the United States Pharmacopeia and National Formulary (USP–NF). Three thousand tons of *Arthrospira platensis* biomass are being produced for food and cosmetics, and it is considered by the UNESCO as the Food of the Millennium. Among the different beneficial health properties of *Arthrospira platensis* biomass consumption are antiviral, anti-inflammatory, and antimicrobial activities, and a positive effect on the immune system, both in animals and humans [3,8]. *Arthrospira platensis* biomass is also used for weight control, and to treat disorders of intestinal flora, cancer, hepatotoxicity, cardiovascular diseases, allergies, anemia, hyperglycemia, hyperlipidemia. *Arthrospira platensis* biomass does not show acute or chronic toxicities, being safe for human consumption [3,8].

Frequently employed extraction methods are solvent extraction, including supercritical CO_2_, molecular distillation, and Soxhlet [9]. In order to increase the extraction yields, physical, chemical, or enzymatic methods can be used beforehand to disrupt the biomass integrity. Methods used for cell disruption might be ultrasonication, microwaving, osmotic shock, freeze-defreeze, homogenization, pressurized liquids, traditional milling, chemical treatments such as soaking in ethanol and water, and enzyme digestion [10,11,12]. Among them, enzyme-assisted extraction can give rise to a great diversity of secondary metabolites, obtained by submitting the biomass to the degrading action of different types of enzymes [8]. The enzyme treatment is applied directly to the biomass before its extraction. This method has been widely studied for the production and conversion of microalgae oil to biodiesel. In contrast, for identification of bioactive products, most of the extracts investigated have been enzyme hydrolysates of microalgae extracts [3,12].

Applied biocatalysis has great potential in this respect, considering the great diversity of biological functions and catalytic mechanisms of natural enzymes. Furthermore, new bioactive compounds can be then obtained via selective enzymatic degradation of these biomasses or their extracts [12,13]. Remarkably, different extract compositions are obtained by directly degrading the biomass or just by degradation of a given extract. Moreover, an efficient enzyme degradation of biomass should permit a more complete extraction of biocomponents. Some biocomponents could not be extracted using other physico-chemical degradation methods of biomass.

Lately, the number of scientific reports of enzyme-assisted extraction studies of *Arthrospira platensis* and microalgae has notably increased. Very often, they describe studies under single extraction conditions, which do not necessarily correspond to the optimal ones for each particular enzyme-extraction system. Not only the extraction yield, but also the proportion and composition of the extract obtained might significantly vary when the extraction conditions are modified [14,15,16]. Hence, there are a lack of systematic studies of enzyme-assisted extraction to determine their optimal conditions to achieve the best bioactive extracts. These studies and the precise identification of peptides formed via proteomic analyses, together with in silico analysis and chemical synthesis of potential peptides [12,17], will provide the necessary complementary knowledge for a further implementation of new therapeutics [18].

Although *Arthrospira platensis* has a very high protein content, this is not always easily assimilated because of some organisms’ disorders. Since assimilation of *Arthrospira platensis* biocomponents depends on how easily they are degraded by organisms, a selective preparation of *Arthrospira platensis* biomass hydrolysates with high peptide and amino acid content, as a result of the enzymatic degradation of biomass, is of great interest at present. In addition, different hydrolysate products with interesting bioactive properties (peptides, amino acids, vitamins, antioxidants, etc.) can be obtained with different selective hydrolytic enzymes [19]. Thus, new extraction processes after degradation of *Arthrospira platensis* biomass by natural enzyme preparations can be implemented for preparation of extracts with a high content of metabolites of easy assimilation [8,20]. These extracts can be used as food supplements with important biologic functions. Different types of biomass digestion by enzymes will give rise to different extracts with unique compositions, which is a function of both the biocatalyst used and the bioprocess conditions.

The anticholesterolemic effect of *Arthrospira platensis* biomass is mainly attributed to the pigment C-phycocyanin [21], which was earlier extracted from biomass [22]. Additionally, the composition of *Arthrospira platensis* makes it a good biomass candidate for selective production of selectively formed secondary metabolites though an enzyme-assisted extraction process. To date, Zhang and Zhang obtained a peptide extract with antitumor activity, via a multiple enzymatic digestion of proteins previously extracted from *Arthrospira platensis* biomass, using freeze-thawing plus ultra-sonication extraction [23]. Hydrolysis was carried out with four proteolytic enzymes (trypsin, alcalase, papain, and pepsin). There is another method permitted to obtain the peptide Ile-Gln-Pro with anti-hypertensive activity [24], which is via proteolysis after submitting the biomass to freeze-thawing plus ultra-sonication. Peptides with antioxidant activity were also obtained from *Arthrospira platensis* [25], this time via direct hydrolysis of cyanobacteria biomass with protease K, followed by peptide extraction by ultracentrifugation and gel chromatography.

*Arthrospira platensis* has natural health effects and a hydrophilic extract of *Arthrospira platensis* biomass, that improves the vasodilator function of the aorta from spontaneously hypertensive rats, has been lately reported [26]. Due to the great potential of *Arthrospira platensis* as a source and/or precursor of new functional products, it is worth it to investigate the different extracts that can be obtained after submitting the biomass to diverse enzyme degradation treatments. In fact, we recently investigated the optimization of four different individual enzyme degradation pretreatments of biomass and the effect of the most important parameters of these processes in the preparation of different hydrophilic *Arthrospira platensis* extracts [16]. In this study, we have investigated the potential of applied biocatalysis for obtaining different secondary metabolites from *Arthrospira platensis* sp. biomass, using these four different enzyme-assisted extraction procedures. The chemical compositions and a variety of bioactivities of the four different enzyme-assisted extracts of *Arthrospira platensis* were comparatively investigated with those of the extract obtained without any prior enzyme digestion. This work describes the different multicomponent (amino acids, proteins, phenols, carbohydrates, oligoelements) and multifunctional *Arthrospira platensis* extracts of secondary metabolites obtained with these four different enzymes under their respective optimal extraction conditions. An *Arthrospira platensis* extract with enhanced properties and interest for the food, cosmetic, and pharmaceutical industries is reported.

## 2. Results and Discussion

In this work, the four different extracts obtained with four distinct enzyme pretreatments of the biomass prior to its solvent extraction were investigated and compared with those of the extract obtained without any prior enzyme digestion.

The weight yield of the lyophilized extracts obtained under their respective optimal conditions (Section 3.2) [16] were determined with respect to the weight of dry biomass submitted to extraction. The weight yields of the lyophilized *Arthrospira platensis* extracts obtained without any enzyme pretreatment (control extract) and using Alcalase^®^, Flavourzyme^®^, Ultraflo^®^, and Vinoflow^®^ were 19.20 ± 0.20% (*w*/*w*), 36.50 ± 0.10% (*w*/*w*), 31.80 ± 0.10% (*w*/*w*), 19.70 ± 0.10% (*w*/*w*), and 26.30 ± 0.10% (*w*/*w*), respectively. Three of the four enzyme digestion methods assayed increased the weight yield of the aqueous phase extract, with proteolytic treatment with Alcalase^®^ being the method that gave the highest yield [16]. The two proteases gave higher yields than the *endo*- and *exo*-glucosidases. These results agree with the fact that the bulk weight of dry *Arthrospira platensis* is predominantly attributed to proteins, with both carbohydrates and lipids as minor components [3]. The best enzyme-assisted method (1% *v*/*w* Alcalase^®^, pH = 6.5, 30 °C and 24 h) gave a 90% (*w*/*w*) increase in the amount of material extracted with respect to the control (simple solvent extraction without prior enzyme digestion of biomass; *p* ≤ 0.0005); that is, a 1.9 times increased yield.

The four different enzyme-assisted extracts obtained in this work under their respective optimal extraction conditions [16] were characterized, and their composition and bioactivities were compared. The enzyme-assisted extracts, as well as the control extract (the same solvent extraction without any enzyme pretreatment of the biomass), should contain different hydrophilic biocomponents [16].

### 2.1. Amino Acid Composition

**Figure 1:**
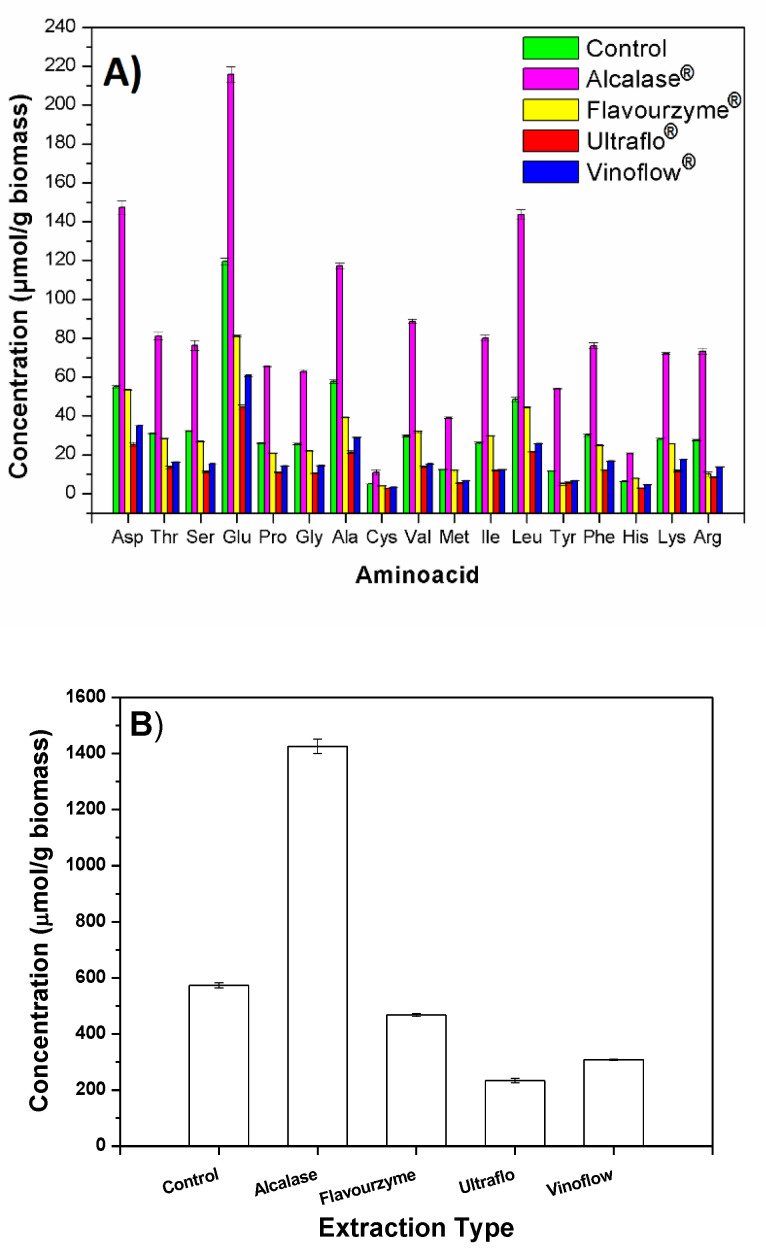
Amino acid content values obtained with different extraction methods in their optimal operation conditions. All amino acid content values were not equal in the *t*-test analyses for a confidence level of 95% (*p* ≤ 0.05), except Met in Flavourzyme^®^ vs. Control extracts, Ile in Ultraflo^®^ vs. Vinoflow^®^, Tyr and Arg in Flavouzyme^®^ vs. Ultraflo^®^. (**A**) Content values for each individual amino acid; (**B**) total amino acid content.

The results of the quantitative analyses are represented in Figure 1.

The extract obtained with Alcalase^®^ had a higher total amino acid content (45% *w*/*w* lyophilized extract) than the control extract (34% *w*/*w*; *p* = 0.0005) and higher than the extracts obtained with the other enzymes (13–17% *w*/*w*; (*p* ≤ 0.0005). Moreover, considering the corresponding weight yields of the extracts (higher in the case of Alcalase^®^ than in the rest), Alcalase^®^ extraction permits the obtention of higher amounts of Glu, Asp, Ala, and Leu, per gram of biomass, compared with other amino acids (117–216 µmol/g biomass (*p* ≤ 0.0005; Figure 1A). Remarkably, the increased recovery of a mole of Tyr per gram of biomass was 4.6–10.8 times higher than with the other methods (*p* ≤ 0.0005). Alcalase^®^ extraction method allowed a recovery of 7.2 and 10.8 times higher mole amounts of Arg and Tyr, respectively, than with the other protease (*p* ≤ 0.0004). For the rest of the amino acids, Alcalase^®^ method was 2.6–3.3 times superior than the other protease method (Flavourzyme^®^, Figure 1B) (*p* ≤ 0.0005).

The extract obtained without any prior enzyme degradation of the biomass (control) as well as those obtained using Ultraflo^®^ and Vinoflow^®^ (non-proteolytic enzymes) contained significant amounts of all amino acids. Their protein material should come from degraded glyco- and lipoproteins of the cyanobacteria. In the case of the control extract, as well as when one submits the biomass to a chemical or physical disruption [11,12,16], the biocomponents might be extracted in different degradation degrees. Due to the presence of natural enzymes of *Arthrospira platensis*, extracts obtained with non-proteolytic enzymes and those directly obtained from physical or chemically disrupted biomass, contain protein material. However, the extracts obtained from digested biomass with different proteolytic enzymes, should have different compositions of peptides and free amino acids than the former ones. In that case, extracts can be formed as a result of the complementary and/or predominant action of the specific enzyme used prior to extraction. This fact should be reflected in both the corresponding weight yields and in the protein composition of the extracts herein studied. Particularly, as a result of an efficient biomass proteolysis, increased amounts of protein material should be extracted. The results obtained with Alcalase^®^ are in good agreement with these considerations, but the use of Flavourzyme^®^ did not significantly improve the extraction efficiency in terms of mole yield of extracted amino acids with respect to the control (Figure 1B).

### 2.2. Peptide Composition

Different peptide sequences were detected in the distinct *Arthrospira platensis* extracts (Table 1). Alcalase^®^ extract contained eight different peptides from those identified in the other extracts (enzymatic or control). This extract contains eight families of exclusive peptides, corresponding to different proteins of *Arthrospira* sp., with their corresponding biological activities, for instance: MKKIEAIIRPF, (nitrogen regulatory protein P-II [*Arthrospira platensis* NIES-39]); ALAVGIGSIGPGLGQGQ, (AtpH [*Arthrospira platensis* HN01]); TTAASVIAAA, (ATP synthase c chain [*Arthrospira platensis* NIES-39]); DFPGDDIPIVS, (Full = Elongation factor Tu; Short = EF-Tu). Several of these peptides were fragments of ATP synthase.

By contrast, 12 peptides were identified in the control extract. Some of them corresponded to fragments of chlorophyll-binding protein and phycocyanin (responsible for photosynthetic and antioxidant activities), others were fragments of phosphoenolpyruvate synthase.

Analyses using *Arthrospira platensis* databases revealed that in all the extracts, there is a significant presence of peptides corresponding to diverse phycocyanin fragments (last column in Table 1). Phycocyanin has important antioxidant activity [22] and these peptides can be responsible for part or all of the antioxidant activity of the extract.

Notably, the peptide sequence Ile-Gln-Pro with anti-hypertensive activity [24], the ACE inhibitor peptide IRDLDYY [27] and other antitumor [23] and bioactive peptides [20], previously obtained by alternative extraction methods, were not detected in this study. Another distinct ACE-1 inhibitory peptide (IAPG) was recently obtained from *Arthrospira platensis* biomass by the combined use of an enzymatic treatment, in silico analysis, and chemical synthesis methods [17]. This fact must be due to the different protocols used for the biomass treatment before extraction of biocomponents by solvents, and supports the great potential of selective enzyme degradations of microalgae biomass to obtain high value secondary metabolites, such as specific peptide sequences.

In a recent study, *Arthrospira platensis* extracts were submitted to different enzyme (papain, pepsin, ficin, and Alcalase) treatments, revealing that papain and ficin release higher amounts of bioactive (antioxidant, antihypertensive (ACE-I and renin), and antidiabetic) peptides. The structures of peptides of these enzyme hydrolysates remain unknown [12]. However, Alcalase^®^ extract described in this work might also include other different peptides. In that case, neither these peptides nor their precursor protein (as well as other bioactive components) would be extracted by the ultrasound and high-pressure homogenization methods utilized by Villaró et al. [12]. By contrast, they will originate during the biomass degradation by the enzyme, and they can further be extracted with the rest of the extractable biocomponents. Once more, the composition and properties of extracts obtained would vary from one production procedure to another. Alcalase^®^ extract herein described was obtained under optimal conditions to degrade this cyanobacteria biomass, as was previously determined [16]. By similar reasoning, one could explain that the three novel antioxidant peptides, recently identified in a pancreatin hydrolysate of C-phycocyanin (MHLWAAK; MAQAAEYYR; MDYYFEER), are not coincident with those obtained in this study [28].

### 2.3. Total Carbohydrate Content of Extracts

**Figure 2:**
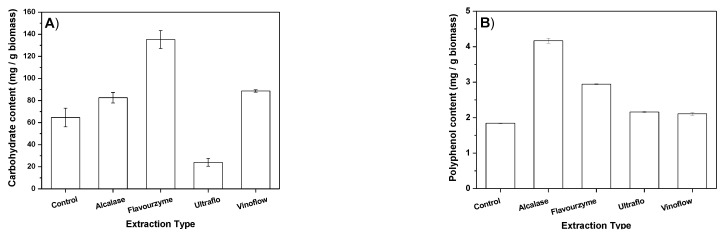
(**A**) Total carbohydrate content values of the extracts. All carbohydrate content values resulted in being not equal in the *t*-test analyses for a confidence level of 95% (*p* ≤ 0.05). (**B**) Total phenols extracted from *Arthrospira platensis* with enzymatic and control (without enzyme) methods. All phenol content values resulted in being not equal in the *t*-test analyses for a confidence level of 95% (*p* ≤ 0.05), except Ultraflo^®^ vs. Vinoflow^®^. Conditions: extracts were obtained with different extraction methods in their optimal operation conditions, namely: pH 6.5, 1% *v*/*w* and 30 °C for Alcalase^®^; pH 6.0, 1% *v*/*w* and 30 °C for Flavourzyme^®^; pH 7.0, 1% *v*/*w* and 30 °C for Ultraflo^®^; pH 6.5, 2% *v*/*w* and 40 °C for Vinoflow^®^. Control extract was obtained using milli-Q water instead of the buffer solution of enzyme, at 30 °C, and for 24 h.

Total carbohydrate contents of lyophilized extracts determined by phenol-sulphuric method are represented in Figure 2A. *Arthrospira platensis* had a smaller proportion of carbohydrates than of proteins. Thus, all the extracts were rich in proteins, but not in carbohydrates (Figure 2A). Moreover, the energetic potential of carbohydrates (15.7 kJ·g^−1^) is smaller than those of lipids (37.6 kJ·g^−1^) or proteins (16.7 kJ·g^−1^) [9]. All enzymatic and control processes extracted similar amounts of carbohydrates (*p* ≤ 0.05), included the Alcalase^®^ process, which gave a 1.3 times higher amount extracted than that obtained with the control process (*p* = 0.004). Only the Flavourzyme^®^ method extracted a higher amount of carbohydrates than the other methods (*p* ≤ 0.005). From each gram of dry biomass, the amount extracted with this enzyme resulted 2.1 and 1.6 times higher than that obtained in both control and Alcalase^®^ processes, respectively.

### 2.4. Total Phenol Content of Extracts

Polyphenolic compounds are abundant in *Arthrospira platensis* [29]. Polyphenols are considered to be physiological antioxidants, which have additional biological roles besides their antioxidant functions, such as modification of on-off switches for enzymes, facilitation of receptor binding as a selective ligand or mimic, or cell-cell signaling at concentrations lower than anticipated for antioxidant activity. They promote the glutathione synthesis (the most prevalent endogenous antioxidant), contributing to an increased content of antioxidant molecules present in the body.

The Folin–Ciocalteu method (FCR) determines the amount of total phenols (polyphenols and other antioxidants) [30]. The results obtained with this method for the enzymatic and control extractions are given in Figure 2B. These results indicate that all the enzymatic methods increase the recovery of total phenols from *Arthrospira platensis* biomass. From each gram of *Arthrospira platensis* biomass, Alcalase^®^ method gave 2.3 times more weight of phenols than the control method (*p* ≤ 0.0005), and 1.4–2.1 times more than the other enzymatic methods (*p* ≤ 0.0005), (1.12 times more than with Flavourzyme^®^ method).

The extraction method and conditions used determine the relative proportions of the extract components. In the case of *Arthrospira platensis* and other microalgae, more proteins are extracted by pressurized water, while more phenols and other antioxidants are recovered with pressurized DMSO [11]. In this work, different total phenol contents were also obtained depending on the enzyme utilized. The enzyme digestions herein studied facilitate the phenol recovery, especially the proteolytic ones. Once more, Alcalase^®^ extract contained the highest total phenol content (Figure 2B).

Most microalgae and *Arthrospira platensis* extracts contain polyphenols and other antioxidants. The increase in numbers of health products marketed as health products rich in polyphenols is due to the assumption of the unproven fact of considering polyphenols as physiological antioxidant ingredients. The extraction procedure here described could facilitate the necessary investigation to clarify this matter. Recent studies suggested that the stability of these antioxidants in the intestinal tract may be increased when they are encapsulated in biopolymeric particles. This fact might increase their potential application for pharmaceuticals and functional food products [31].

### 2.5. Elemental Composition of Extracts

All the extracts were essentially free of toxic elements, although Alcalase^®^ extract has reduced content of As and Ni in comparison with the control extract (Table 2). All extracts had variable amounts of K, Mg, Mn, Ca, Cu, and Fe. The control extract seemed to have significantly higher amounts of all these elements than the others, except Fe and Zn. Alcalase^®^ extract was characterized by a Fe content around 8 ppm and 10 ppm of Zn. However, in this extract, the most abundant micronutrients were K, Mg, and Ca (Table 2).

### 2.6. In Vitro Bioactive Properties of Arthrospira platensis sp. Extracts

#### 2.6.1. Anti-Hypertensive Activity

The values obtained in the determinations of anti-hypertensive activities of the enzymatic and control extracts are summarized in Table 3. The lowest IC_50_ value was obtained for Alcalase^®^ extract, indicating that this extract has the highest ACE activity. Alcalase^®^ extract was 3.8 times superior than the control extract (*p* ≤ 0.0005). Not all the enzymatic extracts had higher bioactivities; only Alcalase^®^ extract had higher hypertensive activity and higher amino acid content than the control extract. Alcalase^®^ extract had 7.3 times more anti-hypertensive activity than the control extract, 5.4 times (*p* = 0.007) more than the extract obtained with the other protease (Flavourzyme^®^), and 11.2 and 6.6 times (*p* = 0.006 and 0.001) more activity than that of Vinoflow^®^ and Ultraflo^®^ extracts, respectively.

#### 2.6.2. Anti-Hyperlipidemic Activity: Inhibition of Pancreatic Lipase

Hyperlipidemia affects different metabolic disorders in blood vessels, producing hypertriglyceridemia and/or hypercholesterolemia. This condition gives rise to vascular problems (micro angiopathy, cardiovascular illness, cerebral-vascular, and/or metabolic syndromes).

All the enzymatic extracts exhibited 35–62 times higher inhibitory capacities than the control extract, (*p* ≤ 0.050; Table 4). The two extracts obtained with proteases (Alcalase^®^ and Flavourzyme^®^) had higher inhibitory activities than the control extract; these two were similar in both extracts (*p* ≤ 0.040).

With respect to the extractive processes, the two processes using proteases (Alcalase^®^ and Flavourzyme^®^) extracted higher inhibitory activity units, being 106 (*p* ≤ 0.0005) and 94 (*p* ≤ 0.0005) times higher than that obtained with the control extract, respectively.

#### 2.6.3. Anti-Hyperlipidemic Activity Analyses: Inhibition of Pancreatic Cholesterol Esterase

It is known that *Arthrospira platensis* biomass has anti-cholesterolemic effects [21], due to the presence of C-phycocyanin in cianobacteria [22]. From these analyses, all enzymatic extracts had higher inhibitory activities than the control, where the activity obtained with Alcalase^®^ method was 16 times higher than that obtained without any enzyme assistance (*p* ≤ 0.0005), and 1.1–3.5 times higher than those obtained with the other enzymes (*p* ≤ 0.034; Table 4). Per gram of biomass, the Alcalase^®^ method extracts 26 times more hypo-cholesterolemic products than the extraction method without using any prior enzyme digestion of biomass (control).

#### 2.6.4. Antioxidant Activity

Antioxidant activities of lyophilized extracts were determined by ABTS and ORAC-Fluorescein Assays. Values of total phenol contents by FCR (Figure 2B) are also related to this bioactivity.

##### ABST and ORAC Methods

Results obtained with these methods are expressed by TEAC value (Trolox Equivalent Antioxidant Capacity) in mmol Trolox per g extract (Table 5). All these extracts had antioxidant activity. The Alcalase^®^ process allows the extraction of more activity units than the control extraction. Alcalase^®^ and Ultraflo^®^ extracts had higher values of antioxidant activity units than the other ones by ORAC method. By ORAC, a higher value of antioxidant activity units per gram of *Arthrospira platensis* biomass was extracted with the Alcalase^®^ process than with the other extraction processes (*p* ≤ 0.0005). It was 4.4 times higher than that obtained in the control extraction (*p* ≤ 0.0005), and 1.6 times higher than that obtained by using the other protease (*p* ≤ 0.0005). The amount of antioxidant activity units extracted with Alcalase^®^, determined by ABTS, was 1.5 times greater than that obtained with the control extraction (*p* ≤ 0.0005), and 1.4 times greater than that obtained with Flavourzyme^®^ (*p* ≤ 0.0005).

Only the Alcalase method extracted more antioxidant activity units by the two methods (ABTS and ORAC) and it also extracted more phenolic compounds (Figure 2A). The higher antioxidant activity of Alcalase^®^ extract is related to the higher presence of phenolic components among other antioxidants. The production of peptides with antioxidant activities from *Arthrospira platensis* biomass has been described [25], but none such peptides were identified in these extracts (Table 1).

This cyanobacterium has a soft cell wall, and the simple gastrointestinal digestion promotes the release of interesting bioactive peptide fractions in the organism, so that direct ingestion of *Arthrospira platensis* or the ingestion of a non-enzyme-assisted extract might have beneficial health effects [18,20,26]. However, the results of this investigation suggest that new different *Arthrospira platensis* extracts with important bioactivities can be obtained via specific biomass degradation with different enzymes to those present in the gastrointestinal tract.

Alcalase^®^ extract is a multicomponent and multifunctional product, which contains high quantities of all amino acids and low molecular weight peptides, but also phenolic compounds, polyphenols, carbohydrates, and oligo elements. This extract differs from the other four extracts herein studied in its higher anti-hypertensive, antioxidant, and anti-hyperlipidemic (anti-hypertriglyceridemic and anti-hypercholesterolemic) activities. Bioactive properties of Alcalase^®^ extract are related to the presence of the eight differential peptide sequences detected in the extract, the higher content in polyphenols, and other antioxidants. Preparation of these types of extracts involves diverse advantages, such as the need for smaller doses to achieve a given effect and the subsequent decay of the incidence of possible secondary effects [26]. This extract could be useful to elaborate formulations with anti-hypertensive, antioxidant, and/or anti-hyperlipidemic activities, being of potential applied use in the food, cosmetic, and pharmaceutical industries [26,27].

In general, there are good perspectives for the use of enzymatic tools to obtain a great diversity of bioactive hydrolysates from microalgae and *Arthrospira platensis* biomass that need to be better explored. Moreover, microencapsulation of *Arthrospira platensis* biomass hydrolysates might enhance their potential for nutra- and pharmaceutical uses, since microencapsulation can increase the bioactivity and stability of these hydrolysates in the stomach [31,32]. Alcalase^®^ extract herein described and/or some of its constituents could be good candidates for these applications. Moreover, ongoing isolation studies of these constituents will permit their identification and thus determination of possible synergic effects on the activities of biocomponents of Alcalase^®^ extract.

## 3. Materials and Methods

### 3.1. Materials

Dry biomass from *Arthrospira platensis* sp. was provided by ASN Leader S.L. (Murcia, Spain). The cyanobacteria biomass was a lyophilized dry powder for nutritional use, with a declared composition of 50–65% proteins, 6–7.5% lipids, 18–22% carbo hydrides, 15% minerals, 0.2% fiber, and 390 cal/100 g (unknown analytical methods). It also contained antioxidants and bioactive products such as phycocyanin (600 mg), chlorophyll (45 mg), and other phytonutrients and vitamins. It is rich in Provitamine A (100% *w*/*w*) and B12 (500% *w*/*w*), and also has B1, B2, B3, E, C, F, and K). Moreover, it has a high content in minerals including oligo elements, and is particularly rich in Fe, Mn, and Cu [33].

The solvents used were HPLC grade. Alcalase^®^ 2.4 L FG, Flavourzyme^®^, Ultraflo^®^ L, and Vinoflow^®^ Max A were commercial enzyme preparations kindly donated by Novozymes A/S (Bagsvaerd, Denmark). Alcalase^®^ has a declared activity of 2.4 AU-A/g. Flavourzyme^®^ has at least 1000 LAPU/g (leucine aminopeptidase units/g determined with Leu-pNA), although the manufacturer indicates that it is not the single activity type in this preparation. Ultraflo^®^ L is standardized on the main activity, β-glucanase (45 fungal β-glucanase (FBG) per g). In addition, the enzyme preparation contains approximately 470 Farbe xylanase units (FXU) per g. Vinoflow^®^ Max A has a declared activity of 46 BGXU/mL.

Alcalase^®^ is a commercial preparation of a serine *endo*-peptidase (EC. 3.4.21.62), mainly subtilisin A. Alcalase^®^ hydrolyzes amino esters including heterocyclic amino esters. Flavourzyme^®^ is a peptidase preparation widely and diversely used for protein hydrolysis, with eight enzymes (two aminopeptidases, two dipeptidyl peptidases, three *endo*peptidases, and one α-amylase) [34]. Ultraflo^®^ L is a multicomponent enzyme preparation that contains a β-glucanase (*endo*-1,3(4)-) and a xylanase (*endo*-1,4-) (EC 3.2.1.6 and EC 3.2.1.8). The enzyme preparation is used to hydrolyze polysaccharide gums. Ultraflo^®^ is specially designed to break down cell wall materials in cereals like *beta*-glucan and xylans [35]. The two types of enzymatic activities in Ultraflo^®^ concern cellulases and catalyzing of the hydrolysis of complex sugars in the amorphous regions of the cellular membrane. The mixed β-glucanase/xylanase preparation is also marketed within the European Union as a feed-additive under the name of “Pentopan/Biofeed Plus.” Vinoflow^®^ Max A is a type of β-glucanase (*exo*-1,3-) preparation. It helps to speed up the aging process of wine, reducing contact time and accelerating its clarification. It is added towards the end of alcoholic fermentation or before malolactic fermentation [36]. All these enzyme preparations (Alcalase^®^, Flavourzyme^®^, Ultraflo^®^, and Vinoflow^®^) are Gras-type hydrolases declared by the American Center for Food Safety and Applied Nutrition Food and Drug Administration [37].

A commercial standard amino acid solution from SIGMA was used for the amino acid analyses of the *Arthrospira platensis* extracts (Ref AAS18 amino acid standard).

### 3.2. Enzyme-Assisted Extraction of Arthrospira platensis Biocomponents

Four different enzyme-assisted extractions were carried out using four commercial enzyme preparations, namely Alcalase^®^ 2.4 L FG, Flavourzyme^®^, Ultraflo^®^ L, and Vinoflow^®^ Max A, according with the methodology described by Verdasco-Martín et al. [16]. Thus, cellular degradation studied was via protein or sugar enzymatic hydrolysis. The enzymatic treatment was followed by solvent extraction of biomass with a hexane-isopropanol mixture (3:2, *v*/*v*). The aqueous phase was separated from the oil phase and residual biomass, and then lyophilized. The control extraction was obtained with the same solvent extraction procedure, but carried out without any enzyme assistance (control experiment). *n*-Hexane and 2-propanol can be used in Europe, USA, and other countries as extraction solvents employed during the processing of raw materials, of foodstuffs, of food components, or of food ingredients [38].

Conditions for the extraction processes in their respective optimal conditions were: pH 6.5, 30 °C, and 1% *v*/*w* Alcalase^®^; pH 6.0, 30 °C, and 1% *v*/*w* Flavourzyme^®^; pH 7.0, 30 °C, and 1% *v*/*w* Ultraflo^®^; pH 6.5, 40 °C, and 2% *v*/*w* Vinoflow^®^ [16]. In the control extraction (without prior enzyme digestion of the biomass), milli-Q water was used instead of the enzymatic preparation in a buffer at 30 °C. All biomass pretreatments lasted 24 h. Aqueous extracted phases were lyophilized for 4 days. All the experiments were carried out at least in triplicate.

### 3.3. Analysis and Characterization of Hydrophilic Extracts

#### 3.3.1. Amino Acid Composition

Quantitative analysis of amino acids was carried out in accordance with the procedure developed by Spackman, Moore, and Stein in a Biochrom 30 Series Amino Acid Analyzer, with a reproducibility >0.5 CV at 10 nmol [39]. Biochrom 30 uses the classic methodology for analysis of amino acids based on ion-exchange liquid chromatography and a continuous post-column reaction with ninhydrin to obtain the qualitative and quantitative analysis, with a sensitivity of ∼10 pmol,

Solutions of the different dry extracts (1–2.6 mg/mL) were prepared in triplicate in hydrolysis tubes, which contained a known concentration of norleucine (internal standard). To calibrate the apparatus, three replicates containing a known amount of the commercial standards solution and the internal standard were used. The three tubes of commercial amino acid standards were submitted to the same treatment as the sample ones, to correct possible losses of amino acids by hydrolysis. Subsequently, all tubes were dried in a Speed Vac apparatus.

The hydrolysis tubes containing the extract solutions were placed in their respective hydrolysis glass bottles. An amount of 200 µL of 6N hydrochloric acid (acid catalysts for peptide hydrolysis) and 50 mg phenol (antioxidant agent) were added to the glass bottles. Subsequently, the tubes of hydrolyzed samples were fully dried. Firstly, for rapid removal of most of the liquid, a phase vacuum for 20 s and a purge of inert gas for another 20 s were applied to each bottle. This process was repeated three times before placing them in an oven at 110 °C for 21 h. To remove any trace of condensed phase formed at room temperature after sample removal from the oven, the tubes were submitted to vacuum drying in the Speed Vac. Finally, the hydrolyzed samples and standard were dissolved in a known amount of buffer. The resultant sample solutions were injected in the analyzer.

#### 3.3.2. Peptide Identification by LC ESI-MS MS

The control and the four enzymatic extracts were analyzed by a liquid chromatography spectrometer with an electrospray ionization mass detector in positive ionization mode (LC ESI-MS MS). Samples were cleaned with C18 tips, model ZipTip Pipette Tips C18 (ref. ZTC18S096 of Millipore). An Ultimate 3000 nanoHPLC (Dionex, Sunnyvale, CA, USA) coupled to an ion trap mass spectrometer AmaZon Speed (Bruker Daltonics, Bremen, Germany) was used for these analyses. An Acclaim C18 PepMap of 75 µm × 15 cm, 3 µm particle size, and 100 Å pore size (ThermoScientific, Waltham, MA, USA) was the reversed phase analytic column employed. The A C18 PepMap of 5 µm particle diameter with 100 Å pore size was used as the trap column in series with the analytical column. A solution of 0.1% trifluoroacetic acid in 98% water/2% acetonitrile solution (ScharLab, Barcelona, Spain) was injected at 3 µL/min. Gradient conditions were obtained with a 0.1% formic acid (Fluka, Buchs, Switzerland) in water (phase A), and 0.1% formic acid in 80% acetonitrile/20% water (phase B), with the nano-pump working at 300 nL/min. The elution gradient was as follows: 5 min in isocratic mode with 96% A: 4% B, followed by a linear increase to 40% B in 60 min, then a linear increase to 95% B in 1 min, the next 7 min. in isocratic conditions of 95% B, and then returning to initial conditions in 10 min. Analyses of the extracts started after injection of their solutions (5 µL of 4 µg/µL), with detection at 214 and 280 nm wavelengths. A second analysis was carried out by injection of 5 µL of extracts solutions (10 µg/µL). The LC system was connected to the ion trap spectrometer by a CaptiveSpray source (Bruker Daltonics, Bremen, Germany), operating in positive mode with a capillary voltage set of 1400 V. The automatic data acquisition collected both MS spectra (*m*/*z* 350–1500) and the MS CID spectra of the 8 more abundant ions. For the 10 µg/µL sample analyses, the MS spectra range was 100–1000 *m*/*z*. Isolation of the same *m*/*z* was avoided using the exclusion dynamics for 1 min after its fragmentation.

The Data Analysis 4.1 software (Bruker Daltonics, Bremen, Germany) was employed for peptide identification from MS and MS/MS data of individual fractions of HPLC. Generic Mascot files of MS/MS spectra were analyzed against a database obtained from NCBInr (National Center for Biotechnology Information) with 68,623 entries of proteins from *Arthrospira*. Mascot v.2.6.0 (Matrix Science, London, UK) was used for the database search [40]. The search parameter was oxidized methionine as the modification variable without enzyme restriction. In MS and in MS/MS modes, the tolerance for peptide mass was of 0.3 Da and 0.4 Da, respectively. For both MS and MS/MS spectra, the precision obtained was ≤±0.1–0.2 Da.

Additionally, in the case of the Alcalase^®^ extract, all MS and MSMS spectra were analyzed using the “de novo” tool of the Peaks software (Bioinformatics Solutions, Inc., Waterloo, ON, Canada). This program combines both the unconditioned “de novo” analysis of MSMS spectra with the more conventional search against organism-specific (i.e., *Arthrospira*) sequence databases. The results table only includes MS and MSMS spectra and their corresponding “de novo” interpretations. Only sequence assignations with confidence values equal or superior to 80 have been included, to avoid doubtful sequences. Please note that this approach cannot distinguish the following identities: I and L; K and Q; F and M (ox).

#### 3.3.3. Total Carbohydrates Content

Total carbohydrates content of lyophilized extracts was determined by phenol-sulphuric method [41]. The extracts were dissolved in milli-Q water at a concentration of 0.2 g/L and analyzed in triplicate, giving the results as the calculated mean value with their standard deviations.

#### 3.3.4. Elemental Analyses

Lyophilized extracts (70–80 mg) were digested in Teflon glasses with 6 mL HNO_3_ for 20 min in a Multiwave 3000 microwave ANTON PAAR model, with a program consisting of: starting from 0 to 500 W in 5 min, maintaining 500 W for 10 min, then increasing to 1000 W in 10 min, and maintaining 1000 W for 20 min (maximal temperature program was set at 240 °C and maximal pressure value reached was 60 Bar). After digestion, all acid solutions were diluted to 25 mL, and 0.5 mL of the corresponding solutions was diluted to 10 mL for the semiquantitative analysis. Samples were analyzed by Inductive Coupled Plasma Mass Spectrometry (ICP-MS) in a NexION 300XX apparatus from PerkinElmer, (Madrid, Spain) with a formerly described method [42].

#### 3.3.5. Anti-Hypertensive Activity

In vitro evaluation of the inhibitory activity of angiotensin I converting enzyme (ACE) was carried out according to a protocol previously described [43]. The system renin-angiotensin constitutes the major regulator system of blood pressure in the human body. In this system, ACE transforms the angiotensin I decapeptide into the vasoconstrictor angiotensin II peptide and deactivates the vasodilator bradykinin. Moreover, this enzyme plays an important role in the control of blood pressure. The IC_50_ value (concentration of *Arthrospira platensis* extract necessary to inhibit 50% of the ACE activity) is used to express the in vitro ACE inhibitory activity of the extracts.

ACE activity was determined following the generation of fluorescence emission due to liberation of the fluorescent product *o*-aminobenzoylglycine (Abz-Gly) from the non-fluorescent substrate Abz-Gly-Phe(NO2)-Pro. ACE inhibitory activity of samples was determined in triplicate using a method earlier described [44]. Fluorescence emission was determined every minute for 30 min at the emission and excitation wavelengths of 355 and 405 nm, respectively, in a fluorometric microplate of a Synergy HT apparatus (Biotek, Winooski, VT, USA). The IC_50_ value was determined from the dose–response curves, where the range of protein concentration (0–0.8 mg/mL) was distributed along a logarithmic scale, using the regression fit function to a non-lineal sigmoidal curve in a GraphPad Prism 4.00 (Graphpad Software Inc., San Diego, CA, USA). The results are given as the mean value calculated for the three replicas with their standard deviations.

#### 3.3.6. Anti-Hyperlipidemic Activity: Inhibition of Pancreatic Lipase Assay

The ability of all the lyophilized *Arthrospira platensis* extracts to inhibit the activity of pig pancreatic lipase (PPL) was determined by a method based on the method prior described by Lee et al., but slightly modified [44]. Briefly, a 13.33 mM stock solution of *p*-nitrophenyl buthyrate (PNPB, from Sigma, St. Louis, MO, USA) in acetonitrile was prepared and kept at −20 °C. A fresh solution of PPL was prepared in Milli-Q water (15 mg/mL).

Reactions were carried out with a substrate concentration of 0.4 mM PNPB in 0.061 M Tris-HCl buffer, pH 8.5 at 37 °C for 5 min. The absorbance increase of *p*-nitrophenol liberated in the presence of 1.5 mg/mL PPL was followed for 3 min at 400 nm in a UV-Vis Jasco V-630 Spectrophotometer. The reaction rate was determined under the same conditions, but in the presence of 0.75 mg/mL extract. The inhibitory activity of each extract was defined as the difference between initial reaction rates of the assays in the presence and absence of the extract. The activity value of each extract was determined in triplicate, and the mean values of results are given with their corresponding standard deviations.

#### 3.3.7. Anti-Hyperlipidemic Activity: Inhibition of Pancreatic Cholesterol Esterase

The capacity of extracts to inhibit the enzyme pancreatic cholesterol esterase was determined by the method described by Pietsch and Gütschow [45]. Cholesterol esterase plays an essential role catalyzing the hydrolysis of diet cholesterol esters at the lumen of the small intestine, producing cholesterol. The inhibition capacity of some compounds facilitates the control of bio-disponibility of cholesterol formed by hydrolysis of cholesterol esters [45]. Hence, these products serve to limit the absorption of free cholesterol in the bloodstream.

A fresh stock solution of cholesterol esterase (0.075 mg/mL) in 100 mM sodium phosphate and 100 mM NaCl, pH 7.0 buffer was prepared and kept until use in ice (0 °C). A stock solution of taurocholic acid (TC, 8.6 mM) was prepared in the same buffer solution, and kept at 4 °C until use. The stock solution of PNPB (13.33 mM) was prepared in acetonitrile. For the reaction carried out in the cuvette of the spectrophotometer, the extract (75 μg/mL) was incubated at 25 °C with taurocholic acid (5.16 mM) and PNPB (0.2 mM) in sodium phosphate buffer 0.1 M, 100 mM NaCl at pH 7.0. The reaction was initiated by the addition of PPL (2.5 µg/mL). The absorbance variation of the reaction mixture was followed at 405 nm. The activity value of each extract was determined in triplicate, and the mean values of results are given with their corresponding standard deviations.

#### 3.3.8. Antioxidant Activity

Different types of methods are recommended for better characterization of the antioxidant activity of a given product [46]. They might be compared when data are given in Trolox equivalence antioxidant capacity (TEAC) units, but also other units are commonly used. Methods of analysis of antioxidants are classified in two types: (i) those based on a reaction of transfer of a hydrogen atom (HAT) and (ii) those based on a reaction involving an electronic transfer (ET) [47]. In this work, both methods were used to characterize the antioxidant activities of the studied *Arthrospira platensis* extracts under study. ORAC method was used as a good example of the HAT method. The ET assays used were the determination of total phenols by Folin–Ciocalteu reagent (FCR) and quantification of the ability of antioxidants to sequester a stable cation radical (ABTS^●+^). FCR is used to quantify the reduction potential of antioxidants, while ORAC method permits the quantification of their capacity for peroxide radical sequestration [46].

All *Arthrospira platensis* extracts were analyzed by ABTS, ORAC, and FCR.

##### ABTS Assay

Re et al. method was used to quantify the ability of antioxidants present in the extracts to sequester a stable cation radical (ABTS^●+^) [47]. The preformed monocation radical of 2,29-azinobis-(3-ethylbenzothiazoline-6-sulphonic acid) (ABTS^●+^) is generated by oxidation of ABTS with potassium persulphate and it is reduced in the presence of hydrogen donor antioxidants. The influence of both the antioxidant concentration and the reaction time of inhibition of the absorbance of the cationic radical are considered to determine the antioxidant activity. The results were expressed by the TEAC value in mmol trolox/g extract, as the concentration (mM) of a standard reference solution (Trolox) with an antioxidant capacity equivalent to that of a solution (1 mM) of the investigated analyte.

##### Hydroxyl Radical Scavenging Assay (ORAC-Fluorescein Assay)

The method described by Dávalos et al. was employed [48]. A hydroxyl radical (OH^●^) is generated in a living organism, having important negative effects in inflammatory processes of tissue illnesses related to oxidative stress.

For ABTS and ORAC analyses, a multimodal plate reader SynergyTM HT with an automatic dispenser of samples, and temperature control from Biotek Instruments (Winooski, VT, USA) was used. The software Biotek Gen5TM was used for data analysis. Each plate with 96 wells was analyzed in quadruplicate, with four standard levels of calibration and 8 repetitions for blank or control. The reaction was started by the automatic addition of 60 μL of ABTS radical or AAPH to the sample solution for ABTS and ORAC assays, respectively. Antiradical activity with ABTS was determined after 10 min of reaction. For ORAC method, the value was read after 180 min of reaction. ABTS activity was determined in quadruplicate and ORAC activity in triplicate.

##### Total Phenol Content by FCR

The Folin–Ciocalteu method that determines the content of polyphenols and other antioxidants was used [30]. This method version reduces by 15% the interference of ascorbic acid.

Prior to analyses, all lyophilized extracts were dissolved in 95% (*v*/*v*) methanol/water (10 g/L), and sonicated (20 kHz) for 10 min to achieve the complete dissolution of the extract. Results are expressed as the mean value of data obtained in three replicas with the corresponding standard deviation.

#### 3.3.9. Statistical Analyses

The experiments were carried out in triplicate or quadruplicate, reporting the results as their corresponding mean value with their standard error which were compared at confidence level of 95% (*p* ≤ 0.05) using the SPSS program.

## 4. Conclusions

The potential of enzyme-assisted extractions for obtaining secondary metabolites from a *Arthrospira platensis* sp. biomass has been investigated. Four different enzyme-assisted extraction methods of *Arthrospira platensis* biocomponents have been optimized and compared in their respective extraction yields and in vitro bioactivities. They have oligo elements among other minerals, vitamins, peptides, amino acids, and antioxidants. These components are essential for maintaining a healthy life, and diverse functional problems are associated with their lack of or deficient presence. Moreover, the extracts exhibit in vitro therapeutic/prophylactic properties, suggesting their potential application in functional feeding and medicine.

Alcalase^®^ extract has superior in vitro anti-hypertensive, antioxidant, and anti-hyperlipidemic (anti-hypertriglyceridemic and anti-hypercholesterolemic) activities to the other extracts. Alcalase^®^ extract obtained by the method herein described has the potential for use in animal and human health care.

Future work needs to be done for isolation and characterization of most relevant bioactive components of Alcalase^®^ extract. These works are in due course in our laboratory. Obtention of the isolated components will permit the investigation of not only their individual properties, but also possible synergistic effects among the extracts components herein described, and/or with other bioactive products. This fact is of great interest for their pharmaceutical application. Moreover, isolation of antioxidants, such as polyphenols, will permit the study of the potential physiological antioxidant properties, which are being attributed to them in an unprobed manner.

*Arthrospira platensis* extracts with different composition and properties can be prepared using different types of enzymes and extraction conditions. This study is in line with the potential of biocatalytic tools to obtain new bioactive products from micro plankton. Enzyme-assisted extractions and particularly those using Alcalase^®^ have a great potential for obtaining not only *Arthrospira platensis*, but also different microalgae extracts with different interesting biomedical activities.

## 5. Patents

Otero, C.; Verdasco-Martín C.M.; Díaz-Lozano, A. Method for producing an extract with antihypertensive, antihyperlipidemic, and antioxidant properties. WO2019229288 (A1)

## Figures and Tables

**Table 1:** The peptide sequence, retention time (Rt, min.), calculated and experimental molecular mass (Da), and mark or score of the identified peptides in the hydrophilic extracts of *Arthrospira platensis* sp. biomass by LC ESI-MS MS.

Extract	RT (min)	Peptide Sequence (Seq. ID:)	M_r_ (Da, Calc.)	M_exp_ (Da)	MARK	Database
Alcalase^®^	42.83	MKKIEAIIRPF (SEQ ID NO: 1)	1344.8	1344.7	46	gi|291569590, nitrogen regulatory protein P-II [*Arthrospira platensis* NIES-39]
	51.60	LPPL (SEQ ID NO: 2)	438.29	438.26	N.P.	Not assignable to a concrete protein
	52.48	ALAVGIGSIGPGLGQGQ (SEQ ID NO: 3)	1493.82	1493.67	90	gi|146186464, AtpH [*Arthrospira platensis* HN01]; gi|355333248, Chain B Microscopic Rotary Mechanism Of Ion Translocation In The F0 Complex Of Atp Synthases; gi|310689674, Chain E Microscopic Rotary Mechanism Of Ion Translocation In The F0 Complex Of Atp Synthases; gi|375325268, ATP synthase subunit C membrane-bound F0 sector; DCCD-binding [*Arthrospira* sp. PCC 8005]
	53.79	TTAASVIAAAL (SEQ ID NO: 4)	987.56	987.44	40	gi|291566395, ATP synthase c chain [*Arthrospira platensis* NIES-39]
	54.56	DFPGDDIPIVS (SEQ ID NO: 5)	1173.56	1173.52	40	gi|119213EFTU_ARTPT, RecName: Full = Elongation factor Tu; Short = EF-Tu
	54.90	LELL (SEQ ID NO: 6)	486.31	486.29	N.P.	Not assignable to a concrete protein
	48.10	WKLLP (SEQ ID NO: 7)	655.40		Dnovo *	
	48.97	CHLLLSM (+15.99) (SEQ ID NO: 8)	831.40		Dnovo *	
Flavourzyme^®^	38.91	RYLSPGELDRIK (SEQ ID NO: 9)	1445.8	1445.6	40	gi|3914334PHAA_ARTPT, RecName: Full = Allophycocyanin alpha chain
	41.54	STEIQVAFGR (SEQ ID NO 10)	1106.57	1106.41	54	gi|14549167PHCA_ARTP, RecName: Full = C-phycocyanin alpha chain
	42.75	IIKEAGNQLF (SEQ ID NO: 11)	1131.63	1131.40	52	gi|3914334PHAA_ARTPT, RecName: Full = Allophycocyanin alpha chain
	43.76	QDAITSVIN (SEQ ID NO: 12)	959.49	959.34	36	gi|3914335APCB_ARTPT, RecName: Full = Allophycocyanin beta chain
	44.41	KTPLTEAVSIA (SEQ ID NO: 13)	1128.64	1128.47	35	gi|60390269PHCA_ARTF, RecName: Full = C-phycocyanin alpha chain
	44.41	VAPEAATDAAGNLL (SEQ ID NO: 14)	1311.67	1311.44	65	gi|375329456, Rec Name: Hemolysin-type calcium-binding region (fragment) [*Arthrospira* sp. PCC 8005]
	46.47	APEAATDAAGNLL (SEQ ID NO: 15)	1212.6	1212.5	78	gi|375329456, Rec Name: Hemolysin-type calcium-binding region (fragment) [*Arthrospira* sp. PCC 8005]
	48.09	MKTPLTEAVSIA (SEQ ID NO: 16)	1259.68	1259.55	40	gi|60390269PHCA_ARTF, RecName: Full = C-phycocyanin alpha chain
	59.20	GAGATFPAPIF (SEQ ID NO: 17)	1047.49	1047.54	42	gi|291565831, putative phosphate ABC transport substrate-binding protein [*Arthrospira platensis* NIES-39]
Ultraflo^®^	42.90	IEEIGVVGVR (SEQ ID NO: 18)	1069.61	1069.54	55	gi|3914334PHAA_ARTPT, RecName: Full = Allophycocyanin alpha chain
	46.90	EIIAGEILDSR (SEQ ID NO: 19)	1214.65	1214.59	41	gi|291567878, enolase [*Arthrospira platensis* NIES-39]
	47.70	GIVDVPLVGGK (SEQ ID NO: 20)	1052.62	1052.53	42	gi|585303353, phosphoenolpyruvate synthase [*Arthrospira* sp. PCC 8005]
	47.70	VVDIKFPDGKLP (SEQ ID NO:21)	1326.75	1326.78	45	gi|291568724, ATP synthase beta chain [*Arthrospira platensis* NIES-39]
	48.08	DVNETVLDNLPK (SEQ ID NO: 22)	1355.69	1355.57	74	gi|406714893, phosphoenolpyruvate synthase [*Arthrospira platensis* C1]
	49.93	GPPLDIKL (SEQ ID NO: 23)	851.51	851.56	30	gi|495331734, chlorophyll a/b binding light-harvesting protein [*Arthrospira* sp. PCC 8005]
	50.90	IDMPGTWQHL (SEQ ID NO: 24)	1196.56	1196.52	44	gi|291568830, glutamate ammonia ligase, glutamine synthetase type I [*Arthrospira platensis* NIES-39]
	58.35	VEVNVSPDIP (SEQ ID NO: 25)	1068.53	1068.50	57	gi|406711236, hypothetical protein SPLC1_S530280 [Arthrospira platensis C1]
	66.58	DSVLFDGNLLP (SEQ ID NO: 26)	1188.60	1188.56	42	gi|809067762, hypothetical protein [*Arthrospira* sp. TJSD091]
Vinoflow^®^	37.20	SNASTIVSNAAR (SEQ ID NO: 27)	1189.61	1189.53	95	gi|37935791, phycocyanin beta subunit, partial [*Arthrospira platensis* FACHB-439]
	38.60	IGVVGVR (SEQ ID NO: 28)	698.44	698.48	49	gi|3914334PHAA_ARTPT, RecName: Full = Allophycocyanin alpha chain
	45.40	IEEIGVVGVR (SEQ ID NO: 29)	1069.61	1069.71	50	gi|3914334PHAA_ARTPT, RecName: Full = Allophycocyanin alpha chain
	56.60	MVAGADDINI (SEQ ID NO: 30)	1017.48	1017.58	45	gi|1119702219, alpha/beta hydrolase [*Arthrospira platensis* major]
	56.60	DGAVLPILHLN (SEQ ID NO: 31)	1160.66	1160.59	41	gi|291570193, probable phosphoketolase [*Arthrospira platensis* NIES-39]
Control	42.46	NGDPFVGHL (SEQ ID NO: 83)	954.46	954.46	43	gi|291569436, photosystem I reaction center subunit XI [*Arthrospira platensis* NIES-39]
	49.82	VFETGIKVVDL (SEQ ID NO: 84)	1218.69	1218.64	53	gi|291568724, ATP synthase beta chain [*Arthrospira platensis* NIES-39]
	49.83	DFFVDKL (SEQ ID NO: 85)	882.45	882.43	42	gi|291571801, phosphoenolpyruvate synthase [*Arthrospira platensis* NIES-39]
	50.10	GPPLDIKL (SEQ ID NO: 86)	851.51	851.48	37	gi|291565679, iron-stress induced chlorophyll-binding protein [*Arthrospira platensis* NIES-39]
	50.50	DVNETVLDNLPKTRTQI (SEQ ID NO: 87)	1955.03	1954.97	41	gi|209495148, phosphoenolpyruvate synthase [*Arthrospira maxima* CS-328]
	53.41	DVNETVLDNLP (SEQ ID NO: 88)	1227.6	1227.5	73	gi|209495148, phosphoenolpyruvate synthase [*Arthrospira maxima* CS-328]
	56.10	DSLISGAAQAVY (SEQ ID NO: 89)	1193.59	1193.59	37	gi|10302997, phycocyanin alpha subunit, partial [*Arthrospira* sp. Paracas P2]
	57.09	GIGNDPLEIQF (SEQ ID NO: 90)	1201.6	1201.6	57	gi|291565650, phycobilisome core-membrane linker polypeptide [*Arthrospira platensis* NIES-39]
	57.76	GLILLPHLATL (SEQ ID NO: 91)	1159.73	1159.62	46	gi|495331734, chlorophyll a/b binding light-harvesting protein [*Arthrospira* sp. PCC 8005]
	58.80	GLILLPHLA (SEQ ID NO: 92)	945.6	945.6	39	gi|291565679, iron-stress induced chlorophyll-binding protein [*Arthrospira platensis* NIES-39]
	58.90	AVLGAGALFHTF (SEQ ID NO: 93)	1202.64	1202.68	45	gi|291565679, iron-stress induced chlorophyll-binding protein [*Arthrospira platensis* NIES-39]
	60.60	DVNETVLDNLP (SEQ ID NO: 94)	1227.6	1227.6	59	gi|209495148, phosphoenolpyruvate synthase [*Arthrospira maxima* CS-328]

* De novo analyses with a confidence level of 80. A precision of ≤±0.1–0.2 Da was obtained, both for MS and MS/MS spectra.

**Table 2:** Elemental composition of *Arthrospira platensis* sp. extracts by semiquantitative analyses.

	Extract				
	Control	Alcalase^®^	Flavourzyme^®^	Ultraflo^®^	Vinoflow^®^
**Toxic Elements**			**(ppm)**		
Hg	-	-	-	-	0.1
Cd	0.1	0.1	0.1	0.4	0.2
As	3.0	0.7	0.4	2.1	1.1
Ni	2.9	0.7	-	-	-
**Trace Elements**			**(ppm)**		
K	33,037	12,737	14,241	19,150	11,916
Mg	3177	1581	1362	2258	1625
Mn	11.7	5.91	4.6	7.1	8.3
Ca	1576	515	312	442	250
Cu	2.7	0.9	0.6	-	0.4
Fe	4.2	8.0	13	5.0	9.1
Se	-	-	-	3.0	-
Zn	-	11	25	-	7.6

**Table 3:** Anti-hypertensive activity. In vitro evaluation of inhibitor activity values of angiotensin-converting enzyme, ACE.

	IC_50_ (mg/mL)	A Extracted * (mL)	A/A Control **
Alcalase^®^ (protease)	0.050 ± 0.006	7.3 ± 0.9	7.3
Flavourzyme^®^ (protease)	0.236 ± 0.012	1.3 ± 0.1	1.3
Ultraflo^®^ (endoglucanase)	0.178 ± 0.029	1.1 ± 0.2	1.1
Vinoflow^®^ (exoglucanase)	0.404 ± 0.029	0.7 ± 0.0	0.7
Control (without enzyme)	0.189 ± 0.028	1.0 ± 0.2	1.0

* Anti-hypertensive activity extracted from 1 g *Arthrospira platensis* biomass, calculated as the product of the inverse of IC_50_ value by the weight obtained of the corresponding extract from 1 g *Arthrospira platensis* biomass (yield (%)/10 g extracted biomass). All ACE values resulted in being not equal in the *t*-text (*p* < 0.05), except Flavourzyme^®^ vs. Ultraflo^®^ and Ultraflo^®^ vs. Control. ** Ratio of total activity units extracted with the enzymatic and control methods.

**Table 4:** Inhibitory activity of pig pancreatic lipase and cholesterol esterase.

In Vitro Inhibitory Activity of Pig Pancreatic Lipase					
Enzyme	A (Δabs/s) ^a^	Inhibition (%)	A_extract_ (Δabs x L/s. g Extract)	A_extracted_ (Δabs x L/s. g Biomass) ^b^	A/A Control ^c^
Without Inhibitor	0.368 ± 0.008	-	-	-	-
Alcalase^®^ (protease)	0.256 ± 0.007	30.3 ± 1.9	0.149 ± 0.019	54.5 ± 6.8	106
Flavourzyme^®^ (protease)	0.254 ± 0.008	31.1 ± 2.1	0.152 ± 0.020	48.3 ± 6.4	94
Ultraflo^®^ (endoglucanase)	0.282 ± 0.003	20.7 ± 0.7	0.115 ± 0.013	22.6 ± 2.6	44
Vinoflow^®^ (exoglucanase)	0.304 ± 0.009	17.5 ± 2.2	0.085 ± 0.021	22.4 ± 5.6	44
Control (without enzyme)	0.366 ± 0.012	0.5 ± 3.0	0.003 ± 0.011	0.5 ± 2.1	1
**In Vitro Inhibitory Activity of Cholesterol Esterase**					
Without Inhibitor	0.515 ± 0.007	-	-	-	-
Alcalase^®^ (protease)	0.432 ± 0.006	16.0 ± 1.2	1.11 ± 0.17	0.404 ± 0.063	26
Flavourzyme^®^ (protease)	0.439 ± 0.008	14.7 ± 1.6	1.01 ± 0.20	0.322 ± 0.064	21
Ultraflo^®^ (endoglucanase)	0.491 ± 0.006	4.5 ± 1.2	0.32 ± 0.17	0.063 ± 0.034	4
Vinoflow^®^ (exoglucanase)	0.452 ± 0.011	12.2 ± 2.1	0.84 ± 0.24	0.221 ± 0.063	14
Control (without enzyme)	0.509 ± 0.006	1.0 ± 1.2	0.08 ± 0.17	0.015 ± 0.033	1

^a^ Activity units as the absorbance increase per second, with 750 mg/L extract in the case of the lipase inhibition and 75 mg/L extract for cholesterol esterase inhibition in the cuvette. ^b^ All inhibitory activity values resulted in being not equal, except Ultraflo^®^ vs. Vinoflow^®^ in the *t*-test analyses for the lipase inhibition study with a confidence level of 95% (*p* ≤ 0.05). ^c^ Ratio of activity units extracted with the enzymatic and control methods.

**Table 5:** Antioxidant activity determined by ABTS and ORAC methods.

Enzyme	ABTS			ORAC ^a^		
	Extract	Total Extracted	R ^c^	Extract	Total Extracted	R ^c^
	TEAC (µmol/g Extract)	TEAC (µmol/g Biomass) ^b^		TEAC (µmol/g Extract)	TEAC (µmol/g Biomass) ^b^	
Alcalase^®^ (protease)	19.7 ± 0.5	7.19 ± 0.18	1.5	462 ± 19	169 ± 7	4.4
Flavourzyme^®^ (protease)	15.5 ± 0.5	4.93 ± 0.16	1.0	325 ± 14	107 ± 4	2.8
Ultraflo^®^ (endoglucanase)	24.1 ± 0.6	4.75 ± 0.12	1.0	269 ± 12	53 ± 2	1.4
Vinoflow^®^ (exoglucanase)	16.4 ± 0.6	4.31 ± 0.16	0.9	260 ± 5	68 ± 1	1.8
Control (without enzyme)	23.4 ± 0.9	4.78 ± 0.12	1.0	199 ± 16	38 ± 3	1.0

^a^ Values obtained after 180 min. ^b^ All antioxidant activity values resulted in being not equal in the *t*-test analyses for a confidence level of 95% (*p* ≤ 0.05), except Flavourzyme^®^ vs. Ultraflo^®^ and Vinoflow^®^ vs. control in ABTS text. ^c^ Ratio of activity units extracted with a given enzymatic method and with the control (non-enzymatic) process.

## Data Availability

The original data are kept by the authors, as part of a patented work licensed to a private company.
